# Low encoding frequencies accurately quantify cardiac mechanics while minimizing phase wrapping in 2D cine DENSE with through-plane dephasing

**DOI:** 10.1186/1532-429X-17-S1-Q118

**Published:** 2015-02-03

**Authors:** Jonathan D Grabau, Gregory J Wehner, Jonathan D Suever, Christopher M Haggerty, Linyuan Jing, David Powell, Sean M Hamlet, Xiaodong Zhong, Frederick H Epstein, Brandon K Fornwalt

**Affiliations:** 1 Pediatrics, University of Kentucky, Lexington, KY, USA; 2 Biomedical Engineering, University of Kentucky, Lexington, KY, USA; 3 MR R&D Collaborations, Siemens Healthcare, Atlanta, GA, USA; 4 Biomedical Engineering, University of Virgina, Charlottesville, VA, USA

## Background

Displacement Encoding with Stimulated Echoes (DENSE) encodes displacement into the phase of the MR signal to quantify cardiac mechanics. The encoding frequency (k_e_) links myocardial displacement to phase. Studies with 2D cine DENSE have used k_e_ of 0.10 cycles/mm, which is high enough to remove the stimulated anti-echo from the sampled k-space and is partially responsible for dephasing the blood signal. This k_e_ leads to wrapping in the phase images and causes intra-voxel dephasing. With the advent of through-plane dephasing, the unwanted echo can be removed without relying on high k_e_. This may allow the use of lower k_e_ to simplify post-processing and increase SNR. Low k_e_, however, may be less sensitive to displacement and result in inaccurate measures of cardiac mechanics. We hypothesized that k_e_ below 0.10 cycles/mm will 1) provide accurate measures of cardiac mechanics, 2) minimize phase wrapping, 3) dephase the blood signal, and 4) improve SNR.

## Methods

Spiral cine DENSE was obtained on 10 healthy subjects and 5 patients with a history of heart disease on a 3T Siemens Tim Trio. For each subject, a mid-ventricular short-axis slice was acquired 5 times with respiratory navigation using a range of encoding frequencies: 0.02, 0.04, 0.06, 0.08, and 0.10 cycles/mm. The acquisition with 0.10 cycles/mm was repeated to assess inter-test reproducibility. Other DENSE parameters included: 6 spiral interleaves, 2.4x2.4x8 mm voxel size, 1.08/17 ms TE/TR, constant 20° flip angle, CSPAMM echo suppression, and through-plane dephasing of 0.08 cycles/mm. Twist, circumferential strain, and radial strain were compared between acquisitions employing different k_e_ using Bland-Altman analyses, coefficient of variation (CoV), and paired t-tests. The percentage of wrapped pixels in the phase images at end-systole was calculated for each k_e_. The magnitude of the blood pool signal was measured through the cardiac cycle to follow dephasing. SNR was calculated at end-systole to assess intra-voxel dephasing.

## Results

**Figure 1:**
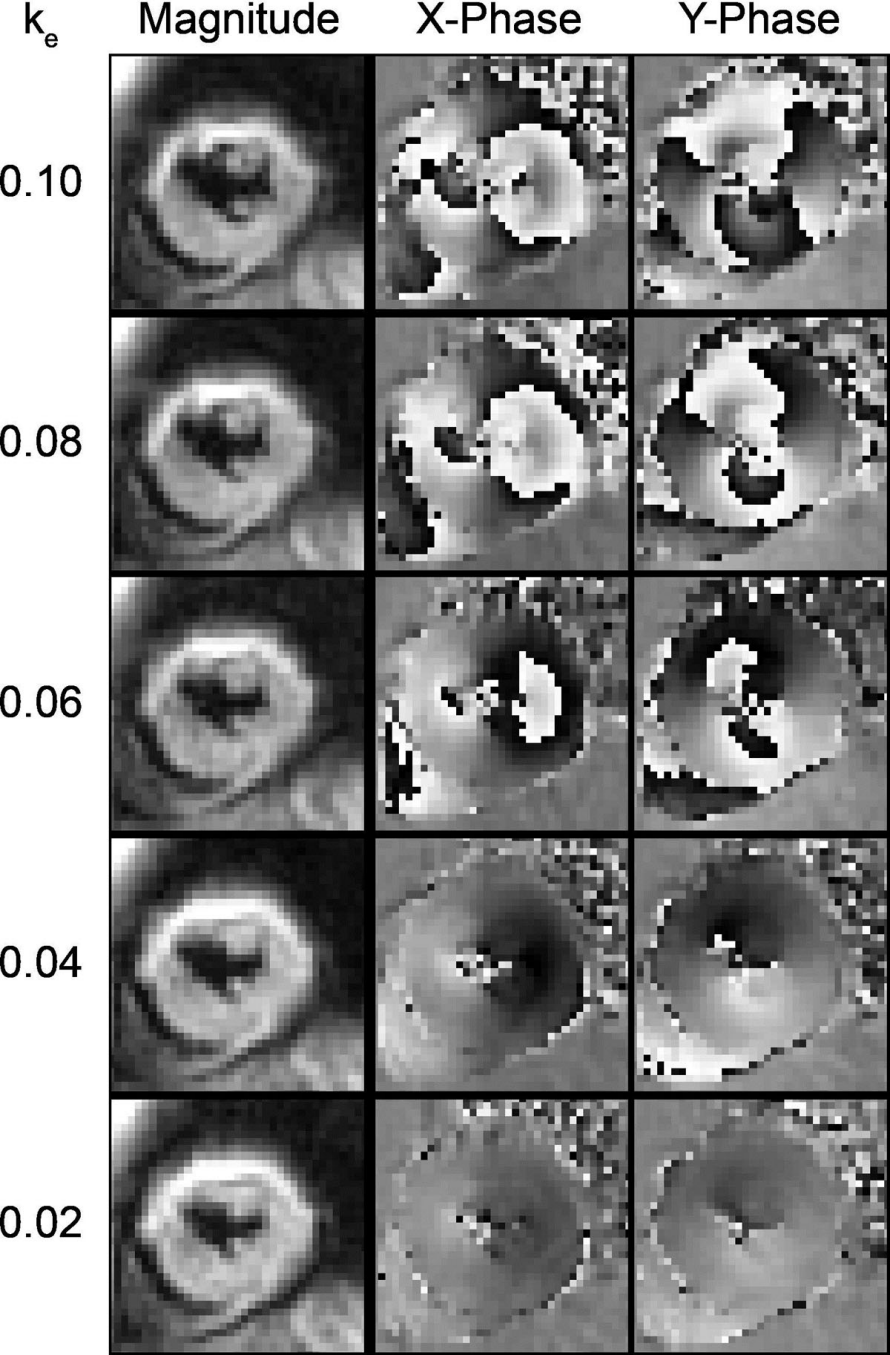
End-systolic, short-axis images from a patient with heart disease. Five separate acquisitions employing different k_e_ yielded unique magnitude and phase images. The prevalence of phase-wrapping in both the x- and y- phase images decreased as k_e_ decreased.

Figure 1 contains end-systolic images from a representative subject. Negligible differences were seen in the strains and twist for all k_e_ between 0.04 and 0.10 cycles/mm (Table 1). These differences were of the same magnitude as the inter-test differences. For 0.02, 0.04, 0.06, 0.08, and 0.10 cycles/mm: 1) the percentage of wrapped pixels in the phase images at end-systole was 0, 0, 6, 19, and 35%, 2) the percent of blood pool signal remaining 68 ms after encoding was 30, 28, 25, 24, and 22%, and 3) the mean SNR at end-systole was 26, 26, 26, 25, and 24, respectively.

**Table 1:** Summary statistics (all 15 subjects) showing good agreement for encoding frequency between 0.04 and 0.10 cycles/mm. Larger biases, 95% LOA, and CoV were observed for encoding frequency of 0.02 cycles/mm.

E(k_e_) *	Circumferential Strain				Radial Strain				Twist (Degrees)			
	
	Bias	95% LOA	Mean CoV	p-value	Bias	95% LOA	Mean CoV	p-value	Bias	95% LOA	Mean CoV	p-value
E(0.10) - E(0.02)	-1.9%	±5.3%	10%	0.02**	3.1%	±21.9%	22%	0.31	-0.50	±1.89	12%	0.07

E(0.10) - E(0.04)	0.0%	±3.2%	4%	1.00	-1.0%	±13.5%	10%	0.57	-0.02	±1.26	7%	0.91

E(0.10) - E(0.06)	0.6%	±2.9%	4%	0.15	0.6%	±11.2%	10%	0.69	-0.19	±0.79	4%	0.09

E(0.10) - E(0.08)	0.3%	±3.0%	4%	0.45	-1.8%	±9.7%	9%	0.19	-0.05	±0.71	4%	0.59

Inter-test	-0.3%	±2.0%	3%	0.23	0.4%	±12.1%	10%	0.81	0.00	±0.93	5%	0.99

*Represents peak strain and twist measured using a particular encoding frequency.

**Statistical significance between peak measures of mechanics using paired-sample t-test at significance level α = 0.05.

***LOA - Limits of Agreement, CoV - Coefficient of Variation.

## Conclusions

For 2D cine DENSE with through-plane dephasing, the encoding frequency can be lowered to 0.04 cycles/mm without causing changes in measures of twist or strain. The amount of wrapping and reliance on un-wrapping algorithms can be substantially reduced with this lower value. The use of lower k_e_ marginally decreases the rate of blood pool dephasing and provides small improvements in SNR.

## Funding

This work was supported by a National Institutes of Health (NIH) Director's Early Independence Award (DP5 OD-012132), NIH grant number T32 HL-072743, and NIH grant numbers UL1TR000117 and KL2 RR033171 from the National Center for Research Resources and the National Center for Advancing Translational Sciences. The content is solely the responsibility of the authors and does not necessarily represent the official views of NIH.

